# Chemical composition of banana meal and rice bran from Australia or South-East Asia

**DOI:** 10.5713/ab.23.0071

**Published:** 2023-05-04

**Authors:** Natalia S. Fanelli, Leidy J. Torres-Mendoza, Jerubella J. Abelilla, Hans H. Stein

**Affiliations:** 1 Division of Nutritional Sciences, University of Illinois, Urbana, IL 61801, USA; 2 DSM Nutritional Products, Mapletree Business City 117440, Singapore

**Keywords:** Alternative Ingredients, Australia, Banana Meal, Chemical Composition, Rice Bran, South-East Asia

## Abstract

**Objective:**

A study was conducted to determine the chemical composition of banana meal and rice bran from Australia or South-East Asia and test the hypothesis that there are no differences in rice bran produced in different countries, but there are differences between full-fat and defatted rice bran.

**Methods:**

Two sources of banana meal and 22 sources of rice bran (full-fat or defatted) from Australia or South-East Asia were used. All samples were analyzed for dry matter, gross energy, nitrogen, amino acids (AA), acid hydrolyzed ether extract (AEE), ash, minerals, total starch, insoluble dietary fiber, and soluble dietary fiber. Banana meal was also analyzed for sugars including glucose, fructose, maltose, sucrose, stachyose, and raffinose.

**Results:**

Chemical analysis demonstrated that banana meal from the Philippines is primarily composed of starch. Full-fat rice bran from Australia had greater (p<0.05) concentrations of AEE, lysine, and glycine than samples from the Philippines and Vietnam. Full-fat rice bran from Australia and Thailand had greater (p<0.05) concentrations of gross energy and most AA than rice bran from Vietnam. Full-fat rice bran from Australia had greater (p<0.05) concentrations of tryptophan and manganese than all other sources, but full-fat rice bran from the Philippines contained less (p<0.05) zinc than all other sources of rice bran. Gross energy, AEE, and copper were greater (p<0.05) in full-fat rice bran compared with defatted rice bran, but defatted rice bran contained more (p<0.05) crude protein, ash, insoluble dietary fiber, total dietary fiber, AA, and some minerals than full-fat rice bran.

**Conclusion:**

Banana meal is a high-energy source that can be used as an alternative ingredient in livestock diets. Full-fat rice bran from Australia and Thailand contained more concentrations of AEE and AA than samples from the Philippines or Vietnam. Full-fat rice bran had more gross energy and AEE than defatted rice bran, whereas defatted rice bran contained more crude protein, ash, and total dietary fiber.

## INTRODUCTION

Rice is an important agricultural crop in most of Asia and Australia, and rice that does not meet specifications for human consumption is often used in the feeding of livestock [1]. Co-products from the rice milling industry are also available and are one of the most important feed resources in all rice-producing countries [1]. Rice bran, a co-product of the rice milling industry, is the outer bran layer of de-hulled paddy rice that is removed to produce polished rice [1]. Fresh rice bran, known as full-fat rice bran, contains 12% to 24% oil, but because oil in rice bran is easily oxidized, oil is sometimes extracted from the bran by solvent extraction, resulting in production of rice oil and defatted rice bran with an acid hydrolyzed ether extract (AEE) concentration of less than 5% [2]. Rice oil is often used for human consumption, whereas defatted rice bran is used in livestock feeding. Instead of extracting the oil from the rice bran, a heating step, often via extrusion, may be used to inactivate the lipase in the bran and thereby prevent oxidation of the oil. This process usually results in full-fat rice bran that can be used directly in diets fed to animals.

Although rice remains one of the most significant crops produced in regions of Asia, other commodities such as fruits and vegetables are also important. Some fruits, such as bananas, are harvested green and are discarded if they do not meet quality specifications for human consumption [3]. By drying these rejected bananas, a banana meal is produced [4]. Banana meal is abundant in the Philippines, Indonesia, and some Pacific islands, where it is used as an energy source in animal diets [4]. Thus, both rice bran and banana meal are available in many countries in Asia or in Australia, but there is limited information about the chemical composition of these ingredients.

Rice bran and banana meal may be used as alternative ingredients instead of traditional cereal grains in animal diets. However, information about their full chemical composition, including analyzed components that add up to 100%, is not always available. In addition, information about the origin of each ingredient and possible differences among origins are also not available and most studies show data about a specific nutrient or area [1,3,4]. There is, therefore, a need to characterize the composition of banana meal and rice bran produced in Asia or Australia. As a result, the objectives of this study were to determine the chemical composition of banana meal and rice bran that are available in South-East Asia or Australia, and to test the hypothesis that there are no differences among countries in the composition of rice bran, but that the chemical composition of full-fat rice bran is different from that of defatted rice bran.

## MATERIALS AND METHODS

### Description of samples

Rice bran and banana meal samples from suppliers in South-East Asia or Australia were delivered to DSM Nutritional Products, Singapore. The suppliers provided between 100 and 300 grams of each ingredient, which was then shipped to the University of Illinois, Urbana, IL, USA, where most of the chemical analyses were conducted. Samples included 2 sources of banana meal from the Philippines, 19 sources of full-fat rice bran from Australia, Indonesia, the Philippines, Thailand, or Vietnam, and 3 sources of defatted rice bran from Indonesia or Vietnam.

### Chemical analysis

Samples of all ingredients were finely ground through a 0.5 mm screen and analyzed for dry matter (method 930.15) [5] and ash (method 942.05) [5]. Gross energy was analyzed using an isoperibol bomb calorimeter (model 6400; Parr Instruments, Moline, IL, USA). Samples were analyzed for amino acids (AA) (method 982.30 E a, b, and c) [5] on a Hitachi Amino Acid Analyzer (Model L8800; Hitachi High Technologies America Inc., Pleasanton, CA, USA) and nitrogen was analyzed by combustion (method 990.03) [5] using a LECO FP628 Nitrogen Analyzer (LECO Corp., Saint Joseph, MI, USA). Crude protein was calculated as nitrogen×6.25. The AEE was analyzed using 3*N* HCl (method 2003.06; Ankom^HCl^, Ankom Technology, Macedon, NY, USA) followed by crude fat extraction using petroleum ether (Ankom^XT15^; Ankom Technology, USA). Insoluble dietary fiber and soluble dietary fiber were quantified according to method 991.43 [5] using the Ankom^TDF^ Dietary Fiber Analyzer (Ankom Technology, USA). Total dietary fiber was calculated as the sum of insoluble and soluble dietary fiber. Minerals were analyzed (method 985.01 a, b, and c) [5] using inductively coupled plasma-optical emission spectrometry (ICP-OES; Avio 200; PerkinElmer, Waltham, MA, USA). Sample preparation included dry ashing at 600°C for 4 h (method 942.05; 10) [5] and wet digestion with nitric acids (method 3050 B) [6]. Total starch was analyzed using the glucoamylase procedure (method 979.10) [5]. Sugars including glucose, fructose, maltose, sucrose, stachyose, and raffinose were also analyzed in banana meal samples using high-performance liquid chromatography (Dionex App Notes 21 and 92).

### Calculations and statistical analysis

For each analysis of feed ingredients, analyzed proximate components were added and subtracted from the concentration of dry matter in each ingredient to calculate the rest fraction according to the following equations:


$$\text{Rest }{\text{fraction}}_{\text{rice bran}}=[\text{dry matter}-(\text{crude protein}+\text{AEE}+\text{ash}+\text{total dietary fiber}+\text{total starch})].$$

$$\text{Rest }{\text{fraction}}_{\text{banana meal}}=[\text{dry matter}-(\text{crude protein}+\text{AEE}+\text{ash}+\text{total dietary fiber}+\text{total starch}+\text{glucose}+\text{fructose}+\text{maltose}+\text{sucrose}+\text{stachyose}+\text{raffinose})].$$

To allow for statistical comparison, nutrients in all samples were adjusted to a 90% dry matter basis. If two or more samples from each country were available, the coefficient of variation and average of samples within each group of feed ingredient were calculated.

Normality of residues and homogeneity of variances were verified using the UNIVARIATE procedure (SAS 9.4 Institute Inc. Cary, NC, USA). Data were analyzed by analysis of variance using the PROC MIXED procedure in SAS to analyze for differences in rice bran composition among countries and to analyze for differences between full-fat and defatted rice bran. The sample was the experimental unit for all analyses. The country or feed ingredient was the fixed effect, and the sample was the random effect. Means were calculated using the LSMEANS statement in SAS, and when significant, means were separated using the PDIFF option in the MIXED procedure. Results were considered significant at p<0.05.

## RESULTS AND DISCUSSION

### Banana meal

The concentration of most nutrients in the two sources of banana meal (Table 1) was within the range of expected values, but crude protein and ash were greater, and starch was less than published values [3,4,7–9]. The variation in chemical composition among sources of banana meal is due to tropical and subtropical countries having different varieties of banana, as well as differences in climate, soil type, and harvesting time, which may result in different degrees of ripeness of the bananas [10,11]. Processing of the harvested bananas may also be distinct among different sources of banana meal. Therefore, it is possible that the bananas used to produce banana meal used in this study were at a riper stage than the bananas used in previous studies because starch decreases with maturation [11]. Differences between the two banana meal samples also indicate that sample 1 was originated from unpeeled bananas, whereas sample 2 was from peeled bananas because banana peel contributes to greater fiber levels, which dilute the percentage of starch in the sample [12]. The high ash level in both samples indicates soil contamination.

The total analyzed components of banana meal were close to 100%, indicating that most nutrients in these ingredients were accounted for [13]. The high coefficient of variation for some of the analyzed nutrients indicates that the ripeness stage or banana variety influenced the chemical composition of the samples. As an example, total dietary fiber is nearly twice as high in one sample than in the other, particularly insoluble dietary fiber. However, the variation in fiber content is consistent with published values for various banana varieties [4,7]. Glucose and fructose were the sugars analyzed in the greatest concentrations, but maltose and sucrose were also present in one of the samples.

Banana meal, with or without peel, can be fed to all types of livestock. Aside from carbohydrates, banana meal contains a significant amount of minerals, particularly potassium, but is low in sulfur-AA, as demonstrated in this study, which is consistent with previous data [9]. In addition, banana contains polyunsaturated fatty acids (primarily linoleic and linolenic acid) that can act as antioxidants, but it may also contain anti-nutritional factors such as tannins (in the peels), oxalate, mycotoxins, and pesticides [9,11].

Bananas that are in fresh or ensiled forms are more commonly used for ruminants, but banana meal can also be used as a source of starch or a lactose substitute in calf diets [9,14]. Banana meal can also be used to replace cereal grains in diets for growing pigs at a maximum inclusion of 25%, but due to the low protein content, diets containing banana meal must be supplemented with additional AA sources. In addition, high levels of banana meal for pigs may cause a negative impact on growth performance due to unbalanced nutrient composition, palatability issues, or the presence of tannins [9]. In diets for poultry, banana meal should not exceed 10% of the grain content because it may be detrimental to growth performance [15].

### Rice bran

The chemical composition of full-fat rice bran from different countries (Tables 2 and 3) was within the range of published values [16–19], but the concentration of AEE ranged from 9% to 20% indicating that some of the ingredients sold as full-fat rice bran had been partially defatted. Likewise, the chemical composition of defatted rice bran (Table 4) was within the range of published data [16–19]. However, when compared with values from previous studies, defatted rice bran from Indonesia had a slightly greater AEE content and a lower crude protein content.

Samples of full-fat rice bran from Australia had greater (p<0.05) concentrations of AEE, lysine, and glycine compared with samples from the Philippines and Vietnam (Table 5). Full-fat rice bran from Australia and Thailand had greater (p<0.05) concentrations of gross energy, isoleucine, leucine, phenylalanine, threonine, valine, alanine, aspartic acid, proline, and serine than sources of full-fat rice bran from Vietnam. When compared with all other countries, full-fat rice bran from Australia had greater (p<0.05) concentrations of tryptophan and manganese, whereas full-fat rice bran from the Philippines contained less zinc (p<0.05) than all other sources.

The observation that there was variation in chemical composition among samples of full-fat rice bran or defatted rice bran produced in different countries may be a result of differences in growing conditions, rice variety, the nature and quality of the bran due to milling condition processes, or hull contamination [20,21]. Gross energy in full-fat rice bran samples ranged from 3,918 to 4,814 kcal/kg, which likely is a result of the indication that some samples were partially defatted. As a result, many products labeled “rice brans” are mixtures of co-products obtained at various stages of the milling process, resulting in variation in chemical composition. As a consequence, frequent analysis of nutrients in rice bran is needed, especially when using higher levels of rice bran in the diets [1]. Differences in mineral concentration may also be a result of differences in soil mineral concentrations or rate of fertilization.

Economic factors such as agricultural credit, support policies, input availability (fertilizers, seeds, or pesticides), research, and knowledge transfer have an impact on production of rice and may also contribute to differences in the composition of rice bran among countries [22]. As an example, the reason full-fat rice bran from Vietnam may contain more insoluble dietary fiber than samples from other countries may be due to the type of production involved because rice mills in Vietnam sometimes mix rice husk fragments with rice bran, resulting in a greater content of total dietary fiber in these samples [23]. Nevertheless, the analyzed components in the rice co-products included in this study were close to 100%, indicating that all nutrients in these ingredients were accounted for [13].

Full-fat rice bran had greater (p<0.05) concentrations of gross energy, AEE, and copper than defatted rice bran (Table 6). In contrast, defatted rice bran contained more (p<0.05) crude protein, ash, insoluble dietary fiber, total dietary fiber, isoleucine, leucine, methionine, phenylalanine, threonine, tryptophan, valine, alanine, glutamic acid, glycine, proline, serine, magnesium, sodium, manganese, and zinc compared with full-fat rice bran. The difference in energy concentration between full-fat and defatted rice bran is a direct result of the difference in AEE content, and the increased protein and ash concentrations in defatted rice bran is a result of concentration of these nutrients when the fat was removed.

Differences in animal responses to feeding rice bran to livestock are believed to be caused by endogenous components or physical properties such as phytate content, enzyme inhibitors, high fiber content, or oxidative rancidity, which can result in palatability reduction and gastrointestinal disturbances [1]. As demonstrated in this study, rice bran is low in calcium and high in phosphorus, however, approximately 80% of the phosphorus in rice bran is bound to phytate. In general, rice bran contain more phytate-bound phosphorus than any other feed ingredients commonly used in diets for pigs and poultry, and phytate-bound phosphorus is largely unavailable to pigs and poultry [24]. However, the use of microbial phytase in rice bran may release some of the phytate-bound phosphorus and improve the digestibility of phosphorus [25].

The oil in full-fat rice bran can become rancid during storage due to the presence of a lipase that becomes active when the bran is separated from the rice kernel. Rancidity rapidly increases the free fatty acid content, which rapidly oxidizes under normal storage conditions [26]. However, oxidation can be slowed by heating or drying after milling [1]. The oil extracted by solvent extraction to produce defatted rice bran has a high commercial value, particularly in countries where cooking oil demand exceeds supply. Production of rice oil and defatted rice bran is, therefore, preferred by rice mills. Defatted rice bran has longer shelf life and lower mycotoxin problems than full-fat rice bran [26]. However, it is dustier, lower in bulk density, and contains more fiber than full-fat rice bran [27], therefore, livestock producers often prefer full-fat rice bran over defatted rice bran as an alternative ingredient due to its higher energy value [28].

Rice bran is a valuable feed for all types of livestock and can be used to supplement oil in diets for dairy cattle [29]. Rice bran is also used in pig and poultry diets due to its high lysine and methionine content. Inclusion of 20% full-fat or defatted rice bran in weanling pig diets had no effects on average daily gain, but inclusion of 30% of full-fat rice bran increased gain to feed ratio [16], which is likely a result of the high AEE concentration in full-fat rice bran. In growing-finishing pigs, inclusion of 30% of full-fat rice bran improved gain to feed ratio without affecting carcass characteristics, but the addition of defatted rice bran decreased gain to feed ratio [17] reflecting the lower energy concentration in defatted rice bran [28]. When full-fat and defatted rice bran were fed to gestating sows, energy digestibility was greater than when fed to growing gilts, regardless of feed intake levels [30]. In broilers diets, an inclusion level of up to 15% to 20% of full-fat or defatted rice bran may be used [1]. Although the present study did not evaluate the effects of microbial enzymes in diets, the inclusion of microbial xylanase may increase energy utilization in rice bran, allowing for greater levels of rice bran inclusion in non-ruminant diets [28].

## CONCLUSION

Banana meal from the Philippines have less starch and greater levels of protein and ash when compared with banana meals from other studies. Nevertheless, banana meal is an excellent source of energy that can be used as an alternative ingredient in diets for livestock contributing to reduction of the environmental impact of discarded banana waste. Full-fat rice bran from Australia and Thailand had greater concentrations of gross energy, AEE, and most AA compared with full-fat rice bran from the Philippines or Vietnam. Full-fat rice bran contained more gross energy and AEE than defatted rice bran, whereas defatted rice bran contained more crude protein, ash, and total dietary fiber than full-fat rice bran.

## Figures and Tables

**Table 1:** Analyzed nutrient composition of banana meal^1)^

Item (%)	Philippines			

	Sample 1	Sample 2	CV	Average
Dry matter	87.20	87.37	0.14	87.29
Gross energy (kcal/kg)	3,429	3,583	3.11	3,506
Crude protein	7.74	5.77	20.62	6.76
AEE	2.06	1.50	22.25	1.78
Ash	13.82	7.05	45.88	10.44
Carbohydrates				
Total starch	41.80	58.10	23.07	49.95
Insoluble dietary fiber	17.65	9.48	42.59	13.57
Soluble dietary fiber	2.27	1.85	14.42	2.06
Total dietary fiber	19.92	11.33	38.87	15.63
Glucose	0.22	0.52	57.33	0.37
Fructose	0.18	0.70	83.57	0.44
Maltose	0.06	0.36	101.02	0.21
Sucrose	ND	0.26	-	-
Stachyose	ND	ND	-	-
Raffinose	ND	ND	-	-
Rest fraction^2)^	4.20	4.42	-	4.31
Indispensable AA				
Arginine	0.25	0.22	9.03	0.24
Histidine	0.12	0.12	0.00	0.12
Isoleucine	0.29	0.20	25.98	0.25
Leucine	0.44	0.35	16.11	0.40
Lysine	0.27	0.21	17.68	0.24
Methionine	0.09	0.06	28.28	0.08
Phenylalanine	0.28	0.21	20.20	0.25
Threonine	0.29	0.18	33.10	0.24
Tryptophan	0.04	0.04	0.00	0.04
Valine	0.36	0.24	28.28	0.30
Dispensable AA				
Alanine	0.41	0.28	26.64	0.35
Aspartic acid	0.58	0.40	25.98	0.49
Cysteine	0.08	0.06	20.20	0.07
Glutamic acid	0.77	0.66	10.88	0.72
Glycine	0.36	0.24	28.28	0.30
Proline	0.27	0.23	11.31	0.25
Serine	0.25	0.19	19.28	0.22
Tyrosine	0.12	0.12	0.00	0.12
Minerals				
Calcium	0.30	0.23	18.68	0.27
Phosphorus	0.17	0.13	18.86	0.15
Magnesium	0.30	0.23	18.68	0.27
Potassium	1.84	1.26	26.46	1.55
Sodium	0.08	0.03	64.28	0.06
Sulfur	0.06	0.04	28.28	0.05
Copper (mg/kg)	27.78	14.22	45.66	21.00
Iron (mg/kg)	2,218.86	1,818.70	14.02	2,018.78
Manganese (mg/kg)	71.05	53.16	20.37	62.11
Zinc (mg/kg)	24.38	23.65	2.15	24.02

AA, amino acids; AEE, acid hydrolyzed ether extract; CV, coefficient of variation; ND, not detected.

1) Except for dry matter, all values were adjusted to 90% dry matter.

2) Rest fraction = calculated using the following equation: [dry matter − (crude protein + AEE + ash + total dietary fiber + total starch + glucose + fructose + maltose + sucrose + stachyose + raffinose)].

**Table 2:** Analyzed nutrient composition of full-fat rice bran from Australia, Indonesia, the Philippines, and Thailand^1)^

Item (%)	Australia			Indonesia	Philippines				Thailand				
		
	Sample 1	Sample 2	CV		Sample 1	Sample 2	Sample 3	CV	Sample 1	Sample 2	Sample 3	Sample 4	CV
Dry matter	91.82	92.35	0.41	90.89	87.63	89.82	89.94	1.46	91.40	88.70	89.98	88.94	1.37
Gross energy (kcal/kg)	4,615	4,814	2.99	4,335	4,383	4,180	4,612	4.92	4,496	4,610	4,507	4,604	1.34
Crude protein	13.08	15.34	11.27	12.28	11.50	9.93	13.11	13.81	12.85	14.02	12.67	13.92	5.27
AEE	17.02	20.10	11.73	13.97	11.17	10.26	18.37	33.48	15.49	17.23	15.11	16.64	6.11
Ash	7.96	8.33	3.24	8.53	9.41	9.36	9.02	2.28	7.99	8.04	7.02	7.41	6.39
Carbohydrates													
Total starch	27.05	17.93	28.67	27.13	33.48	25.75	26.02	15.44	28.36	23.95	29.01	28.23	8.47
Insoluble dietary fiber	20.49	22.12	5.43	22.68	19.82	29.96	18.11	28.30	21.86	21.61	20.90	19.93	4.09
Soluble dietary fiber	2.06	3.51	36.84	2.57	1.85	2.00	2.70	20.76	4.53	2.54	1.70	2.33	44.11
Total dietary fiber	22.54	25.63	9.06	25.25	21.67	31.96	20.81	25.01	26.39	24.15	22.61	22.26	7.88
Rest fraction^2)^	2.35	2.67	-	2.84	2.76	2.74	2.67	-	−1.07	2.62	3.58	1.54	-
Indispensable AA													
Arginine	0.97	1.18	13.74	0.92	0.78	0.71	1.02	19.34	0.98	0.99	0.94	1.01	3.11
Histidine	0.34	0.44	17.28	0.33	0.31	0.25	0.36	17.89	0.35	0.37	0.34	0.36	3.29
Isoleucine	0.49	0.53	5.03	0.44	0.45	0.37	0.47	12.27	0.49	0.49	0.47	0.50	2.35
Leucine	0.90	1.00	7.57	0.83	0.81	0.67	0.88	13.50	0.91	0.93	0.87	0.93	3.23
Lysine	0.66	0.80	13.83	0.59	0.51	0.44	0.66	20.74	0.62	0.63	0.60	0.63	2.15
Methionine	0.25	0.28	10.07	0.25	0.23	0.20	0.26	13.07	0.27	0.27	0.25	0.27	4.18
Phenylalanine	0.58	0.64	7.51	0.54	0.53	0.42	0.57	15.31	0.59	0.61	0.57	0.61	3.03
Threonine	0.47	0.57	12.94	0.46	0.41	0.35	0.47	14.53	0.47	0.49	0.46	0.49	2.65
Tryptophan	0.08	0.19	57.28	0.08	0.08	0.07	0.09	12.39	0.08	0.08	0.08	0.09	6.80
Valine	0.74	0.82	7.60	0.67	0.66	0.55	0.72	13.28	0.74	0.76	0.71	0.75	2.93
Dispensable AA													
Alanine	0.78	0.92	10.97	0.72	0.66	0.57	0.78	15.68	0.77	0.79	0.74	0.79	3.09
Aspartic acid	1.15	1.36	12.25	1.07	1.02	0.85	1.14	14.42	1.11	1.14	1.07	1.14	2.96
Cysteine	0.28	0.34	12.85	0.27	0.28	0.22	0.30	15.41	0.29	0.30	0.27	0.30	5.63
Glutamic acid	1.67	1.93	10.36	1.56	1.47	1.27	1.68	13.83	1.69	1.72	1.67	1.80	3.31
Glycine	0.69	0.81	11.61	0.63	0.62	0.49	0.68	16.14	0.67	0.70	0.64	0.69	3.87
Proline	0.52	0.60	10.66	0.51	0.48	0.41	0.52	11.78	0.53	0.56	0.52	0.55	3.08
Serine	0.48	0.59	15.03	0.49	0.44	0.37	0.50	14.80	0.50	0.53	0.51	0.54	3.02
Tyrosine	0.32	0.40	14.89	0.34	0.36	0.26	0.39	20.10	0.36	0.39	0.35	0.41	7.44
Minerals													
Calcium	0.05	0.05	0.41	0.04	0.05	0.05	0.05	1.49	0.06	0.06	0.05	0.07	14.18
Phosphorus	1.92	2.05	4.47	1.74	1.47	2.10	1.48	21.59	1.70	1.95	1.75	1.52	10.22
Magnesium	0.68	0.85	15.92	0.68	0.69	0.94	0.62	22.62	0.75	0.71	0.69	0.57	11.58
Potassium	1.61	1.88	11.08	1.40	1.27	1.93	1.30	24.88	1.44	1.38	1.34	1.30	4.43
Sodium	0.01	0.01	0.41	0.01	0.01	0.01	0.01	1.49	0.01	0.01	0.01	0.01	1.36
Sulfur	ND	ND	-	ND	ND	ND	0.05	-	ND	ND	ND	ND	-
Copper (mg/kg)	29.86	32.04	5.00	27.78	25.27	35.16	30.98	16.30	27.25	23.94	26.49	32.46	13.00
Iron (mg/kg)	102.58	145.41	24.43	127.26	102.31	108.34	87.01	11.08	105.95	121.78	100.84	83.35	15.37
Manganese (mg/kg)	226.78	235.17	2.57	128.36	141.50	121.35	119.73	9.51	101.83	122.40	102.33	138.15	15.07
Zinc (mg/kg)	60.52	70.46	10.74	58.43	46.58	48.53	42.63	6.54	62.80	55.98	54.28	60.11	6.65

AA, amino acids; AEE, acid hydrolyzed ether extract; CV, coefficient of variation; ND, not detected.

1) Except for dry matter, all values were adjusted to 90% dry matter.

2) Rest fraction = calculated using the following equation: [dry matter − (crude protein + AEE + ash + total dietary fiber + total starch)].

**Table 3:** Analyzed nutrient composition of full-fat rice bran from Vietnam^1)^

Item (%)	Vietnam									

	Sample 1	Sample 2	Sample 3	Sample 4	Sample 5	Sample 6	Sample 7	Sample 8	Sample 9	CV
Dry matter	90.52	90.38	89.65	90.65	92.48	91.99	91.25	90.75	90.43	0.96
Gross energy (kcal/kg)	4,127	4,608	4,515	4,210	4,094	3,918	4,403	4,173	4,332	5.13
Crude protein	11.37	13.22	13.14	11.89	9.10	10.48	14.27	10.48	12.02	13.74
AEE	13.96	17.37	14.88	13.55	8.98	10.92	13.52	12.75	12.44	17.98
Ash	14.66	9.56	9.15	11.77	8.61	15.64	7.56	11.15	9.42	25.46
Carbohydrates										
Total starch	19.79	16.23	20.58	24.62	28.03	17.22	32.35	20.93	23.39	22.89
Insoluble dietary fiber	25.75	29.67	24.39	22.44	30.95	30.92	15.88	23.01	27.37	19.00
Soluble dietary fiber	1.29	2.39	1.91	2.58	1.07	3.13	2.37	8.73	1.09	86.49
Total dietary fiber	27.04	32.06	26.30	25.02	32.02	34.05	18.25	31.74	28.46	17.22
Rest fraction^2)^	3.18	1.55	5.95	3.15	3.26	1.69	4.05	2.96	4.27	-
Indispensable AA										
Arginine	0.83	0.93	0.80	0.85	0.63	0.75	1.08	0.75	0.88	15.17
Histidine	0.30	0.34	0.34	0.32	0.23	0.28	0.37	0.28	0.32	13.44
Isoleucine	0.41	0.45	0.42	0.44	0.34	0.37	0.53	0.39	0.43	12.98
Leucine	0.75	0.84	0.77	0.80	0.63	0.70	0.99	0.72	0.78	12.73
Lysine	0.55	0.62	0.58	0.56	0.45	0.51	0.61	0.52	0.56	9.72
Methionine	0.21	0.24	0.23	0.24	0.19	0.21	0.28	0.21	0.25	11.37
Phenylalanine	0.49	0.55	0.51	0.52	0.39	0.46	0.64	0.47	0.49	13.75
Threonine	0.40	0.46	0.42	0.42	0.33	0.37	0.48	0.38	0.41	11.24
Tryptophan	0.06	0.08	0.07	0.07	0.06	0.07	0.09	0.07	0.08	13.64
Valine	0.62	0.70	0.65	0.66	0.51	0.57	0.79	0.59	0.64	12.76
Dispensable AA										
Alanine	0.66	0.74	0.67	0.69	0.54	0.61	0.81	0.62	0.66	11.41
Aspartic acid	0.91	1.05	1.00	0.96	0.82	0.91	1.14	0.89	1.02	10.02
Cysteine	0.25	0.30	0.30	0.26	0.20	0.23	0.32	0.24	0.26	14.35
Glutamic acid	1.38	1.55	1.32	1.50	1.20	1.35	1.99	1.40	1.52	15.41
Glycine	0.59	0.65	0.65	0.60	0.47	0.54	0.70	0.54	0.59	12.01
Proline	0.45	0.51	0.49	0.48	0.39	0.43	0.56	0.45	0.48	10.55
Serine	0.43	0.49	0.44	0.45	0.36	0.40	0.55	0.43	0.45	12.13
Tyrosine	0.28	0.33	0.31	0.29	0.22	0.25	0.44	0.27	0.30	20.85
Minerals										
Calcium	0.06	0.06	0.08	0.07	0.08	0.73	0.04	1.61	0.05	173.47
Phosphorus	1.43	2.07	1.92	1.68	1.05	1.28	1.95	1.41	2.53	26.99
Magnesium	0.64	0.76	0.61	0.74	0.49	0.64	0.84	0.58	1.04	23.26
Potassium	1.25	1.50	1.45	1.35	1.01	1.40	1.46	1.23	2.05	20.06
Sodium	0.02	0.01	0.01	0.01	0.01	0.02	0.01	0.01	0.01	35.79
Sulfur	0.01	ND	ND	ND	0.01	0.03	ND	0.07	ND	-
Copper (mg/kg)	33.02	32.25	29.85	27.66	29.84	34.71	27.72	34.25	36.12	9.73
Iron (mg/kg)	167.74	125.61	150.18	903.15	224.54	598.93	133.22	143.51	118.53	97.06
Manganese (mg/kg)	152.02	143.14	161.71	130.41	134.67	146.69	11.11	132.38	130.47	35.25
Zinc (mg/kg)	60.68	66.98	58.41	61.51	46.43	59.17	63.67	51.10	56.08	10.83

AA, amino acids; AEE, acid hydrolyzed ether extract; CV, coefficient of variation; ND, not detected.

1) Except for dry matter, all values were adjusted to 90% dry matter.

2) Rest fraction = calculated using the following equation: [dry matter − (crude protein + AEE + ash + total dietary fiber + total starch)].

**Table 4:** Analyzed nutrient composition of defatted rice bran from Indonesia and Vietnam^1)^

Item (%)	Indonesia	Vietnam			

		Sample 1	Sample 2	CV	Average
Dry matter	86.42	89.05	89.36	0.25	89.21
Gross energy (kcal/kg)	3,832	3,726	3,707	0.36	3,717
Crude protein	13.21	17.20	17.49	1.19	17.35
AEE	4.51	1.46	1.48	1.21	1.47
Ash	10.90	12.63	11.22	8.38	11.93
Carbohydrates					
Total starch	23.54	16.68	23.47	23.92	20.07
Insoluble dietary fiber	34.37	37.39	33.54	7.69	35.47
Soluble dietary fiber	1.87	2.43	2.72	8.07	2.57
Total dietary fiber	36.24	39.82	36.26	6.62	38.04
Rest fraction^2)^	1.60	2.21	0.08	-	1.15
Indispensable AA					
Arginine	0.86	1.08	1.20	7.26	1.14
Histidine	0.32	0.40	0.43	4.87	0.42
Isoleucine	0.46	0.61	0.65	5.41	0.63
Leucine	0.85	1.14	1.26	6.89	1.20
Lysine	0.56	0.77	0.81	3.38	0.79
Methionine	0.24	0.31	0.34	6.28	0.33
Phenylalanine	0.54	0.72	0.80	7.30	0.76
Threonine	0.45	0.60	0.65	6.60	0.63
Tryptophan	0.09	0.15	0.15	0.25	0.15
Valine	0.70	0.92	1.00	5.71	0.96
Dispensable AA					
Alanine	0.71	0.96	1.06	6.83	1.01
Aspartic acid	0.96	1.30	1.41	5.54	1.36
Cysteine	0.26	0.32	0.33	1.93	0.33
Glutamic acid	1.53	2.06	2.26	6.36	2.16
Glycine	0.62	0.83	0.90	5.54	0.86
Proline	0.53	0.68	0.74	5.82	0.71
Serine	0.47	0.62	0.72	10.47	0.67
Tyrosine	0.32	0.37	0.43	10.36	0.40
Minerals					
Calcium	0.05	0.12	0.13	5.41	0.13
Phosphorus	1.95	1.99	2.33	10.99	2.16
Magnesium	0.85	0.89	1.06	12.21	0.97
Potassium	1.64	0.86	1.09	16.61	0.97
Sodium	0.02	0.01	0.02	46.92	0.02
Sulfur	ND	ND	ND	-	0.00
Copper (mg/kg)	38.23	12.98	13.54	2.98	13.26
Iron (mg/kg)	167.76	567.43	524.31	5.59	545.87
Manganese (mg/kg)	131.79	355.54	289.62	14.45	322.58
Zinc (mg/kg)	65.55	122.88	126.29	1.94	124.58

AA, amino acids; AEE, acid hydrolyzed ether extract; CV, coefficient of variation; ND, not detected.

1) Except for dry matter, all values were adjusted to 90% dry matter.

2) Rest fraction = calculated using the following equation: [dry matter − (crude protein + AEE + ash + total dietary fiber + total starch)].

**Table 5:** Chemical composition of full-fat rice bran collected from Australia, the Philippines, Thailand, and Vietnam^1)^

Item (%)	Countries					

	Full-fat rice bran					

	Australia	Philippines	Thailand	Vietnam	SEM	p-value
Dry matter	92.09^a^	89.13^c^	89.76^bc^	90.90^ab^	0.55	0.018
Gross energy (kcal/kg)	4,714^a^	4,392^ab^	4,554^a^	4,264^b^	104.04	0.026
Crude protein	14.21	11.51	13.37	11.77	0.80	0.101
AEE	18.56^a^	13.27^b^	16.12^ab^	13.15^b^	1.40	0.049
Ash	8.15	9.26	7.62	10.84	1.15	0.096
Carbohydrates						
Total starch	22.63	28.80	27.39	22.70	2.69	0.164
Insoluble dietary fiber	20.04	21.74	21.08	25.30	2.45	0.196
Soluble dietary fiber	2.79	2.18	2.78	2.73	1.04	0.973
Total dietary fiber	23.23	24.29	23.85	28.15	2.52	0.243
Rest fraction^2)^	2.51	2.72	1.67	3.34	0.75	0.285
Indispensable AA						
Arginine	1.08	0.84	0.98	0.83	0.07	0.060
Histidine	0.39	0.31	0.36	0.31	0.02	0.079
Isoleucine	0.51^a^	0.43^ab^	0.49^a^	0.42^b^	0.03	0.049
Leucine	0.95^a^	0.79^ab^	0.91^a^	0.78^b^	0.05	0.040
Lysine	0.73^a^	0.54^b^	0.62^ab^	0.55^b^	0.04	0.013
Methionine	0.27	0.23	0.27	0.23	0.01	0.076
Phenylalanine	0.61^a^	0.51^ab^	0.60^a^	0.50^b^	0.03	0.049
Threonine	0.52^a^	0.41^bc^	0.48^ab^	0.41^c^	0.03	0.017
Tryptophan	0.14^a^	0.08^b^	0.08^b^	0.07^b^	0.01	0.033
Valine	0.78^a^	0.64^ab^	0.74^a^	0.64^b^	0.04	0.040
Dispensable AA						
Alanine	0.85^a^	0.67^bc^	0.77^ab^	0.67^c^	0.04	0.023
Aspartic acid	1.26^a^	1.00^bc^	1.12^ab^	0.97^c^	0.06	0.011
Cysteine	0.31	0.27	0.29	0.26	0.02	0.304
Glutamic acid	1.80	1.47	1.72	1.47	0.11	0.080
Glycine	0.75^a^	0.60^b^	0.68^ab^	0.59^b^	0.04	0.038
Proline	0.56^a^	0.47^bc^	0.54^ab^	0.47^c^	0.03	0.037
Serine	0.54^a^	0.44^ab^	0.52^a^	0.44^b^	0.03	0.049
Tyrosine	0.36	0.34	0.38	0.30	0.03	0.159
Minerals						
Calcium	0.30	0.24	0.06	0.37	0.26	0.704
Phosphorus	1.99	1.68	1.73	1.70	0.21	0.808
Magnesium	0.77	0.75	0.68	0.70	0.08	0.878
Potassium	1.75	1.50	1.37	1.41	0.14	0.390
Sodium	0.01	0.01	0.01	0.01	0.00	0.586
Sulfur	0.01	0.02	0.00	0.02	0.01	0.541
Copper (mg/kg)	30.95	30.47	27.54	31.71	1.88	0.292
Iron (mg/kg)	124.00	99.22	102.98	285.05	114.47	0.383
Manganese (mg/kg)	230.98^a^	127.53^b^	116.18^b^	126.96^b^	19.19	0.010
Zinc (mg/kg)	65.49^a^	45.91^b^	58.29^a^	58.23^a^	3.03	0.009

AA, amino acids; AEE, acid hydrolyzed ether extract; SEM, standard error of the means.

1) Except for dry matter, all values were adjusted to 90% dry matter.

2) Rest fraction = calculated using the following equation: [dry matter − (crude protein + AEE + ash + total dietary fiber + total starch)].

a–c Means in a row without a common superscript letter differ (p<0.05).

**Table 6:** Comparison between full-fat and defatted rice bran^1)^

Item (%)	Rice bran			

	Full-fat	Defatted	SEM	p-value
Dry matter	90.50	88.28	0.57	0.012
Gross energy (kcal/kg)	4397	3708	155.25	0.041
Crude protein	12.35	15.79	0.87	0.015
AEE	14.41	2.48	1.25	<0.001
Ash	9.50	12.73	0.63	0.026
Carbohydrates				
Total starch	24.74	21.91	1.23	0.066
Insoluble dietary fiber	23.57	36.65	1.37	0.012
Soluble dietary fiber	2.65	2.28	0.46	0.436
Total dietary fiber	26.22	39.06	1.22	0.006
Rest fraction^2)^	2.78	1.30	0.60	0.094
Indispensable AA				
Arginine	0.89	1.05	0.06	0.101
Histidine	0.33	0.38	0.02	0.173
Isoleucine	0.45	0.57	0.03	0.003
Leucine	0.83	1.08	0.05	0.003
Lysine	0.58	0.71	0.04	0.057
Methionine	0.24	0.29	0.02	0.028
Phenylalanine	0.54	0.69	0.03	0.006
Threonine	0.44	0.56	0.03	0.015
Tryptophan	0.08	0.12	0.01	0.043
Valine	0.68	0.87	0.04	0.003
Dispensable AA				
Alanine	0.71	0.91	0.05	0.007
Aspartic acid	1.04	1.22	0.07	0.090
Cysteine	0.27	0.30	0.02	0.390
Glutamic acid	1.56	1.95	0.10	0.016
Glycine	0.63	0.78	0.04	0.012
Proline	0.50	0.65	0.03	0.003
Serine	0.47	0.60	0.03	0.017
Tyrosine	0.33	0.37	0.04	0.535
Minerals				
Calcium	0.17	0.10	0.16	0.744
Phosphorus	1.74	2.09	0.15	0.108
Magnesium	0.71	0.93	0.06	0.013
Potassium	1.45	1.20	0.12	0.154
Sodium	0.01	0.02	0.00	0.018
Sulfur	0.01	0.00	0.01	0.450
Copper (mg/kg)	30.35	21.58	2.48	0.021
Iron (mg/kg)	192.12	447.68	124.44	0.253
Manganese (mg/kg)	135.80	258.98	24.91	0.002
Zinc (mg/kg)	57.07	104.52	5.94	<0.001

AA, amino acids; AEE, acid hydrolyzed ether extract; SEM, standard error of the means.

1) Except for dry matter, all values were adjusted to 90% dry matter.

2) Rest fraction = calculated using the following equation: [dry matter − (crude protein + AEE + ash + total dietary fiber + total starch)].
